# Structure Analysis of Effective Chemical Compounds against Dengue Viruses Isolated from *Isatis tinctoria*

**DOI:** 10.1155/2018/3217473

**Published:** 2018-04-01

**Authors:** Bo Gao, Jianming Zhang, Lianhui Xie

**Affiliations:** ^1^College of Life Sciences of Fujian Province, Fujian Agriculture and Forestry University, Fuzhou, Fujian 350002, China; ^2^Fujian International Travel Healthcare Center, Fuzhou, Fujian 350001, China; ^3^Clinical Laboratory, The Affiliated Quanzhou First Hospital of Fujian Medical University, No. 248 East Street, Quanzhou, Fujian 362002, China; ^4^Key Laboratory of Plant Virology of Fujian Province, Fujian Agriculture and Forestry University, Fuzhou, Fujian 350002, China

## Abstract

The history of Chinese herb research can be traced back to thousands of years ago, and the abundant knowledge accumulated for these herbs makes them good candidates for developing new natural drugs. *Isatis tinctoria* is probably the most well-studied Chinese herb, which has been identified to be effective against dengue fever. However, the underlying biological mechanisms are still unclear. In this study, we adopt combined methods of bioactive trace technology and phytochemical extraction and separation, to guide the isolation and purification of the effective chemical constituents on the water-soluble components of aerial parts of *Isatis tinctoria*. In addition, we apply polarimetry and 1D or 2D nuclear magnetic resonance (NMR) spectroscopy to identify their structures, which lay a foundation for further study on the biological mechanisms underlying medicinal effects of *Isatis tinctoria* using *in vitro* and *in vivo* experiments. Specifically, we identify and infer the structures of 27 types of chemical compounds named GB-1, GB-2, …, GB-27, respectively, among which GB-7 is a novel compound. Further study of these compounds is critical to reveal the secrets behind the medicinal effects of *Isatis tinctoria*.

## 1. Introduction

Dengue fever (DF) is a kind of acute infectious diseases caused by dengue virus (DV). Its major clinical symptoms include acute high fever, headache, generalized myalgia, ostalgia and arthralgia, rash, hemorrhagic tendency, and low white blood cell count [1]. Dengue hemorrhagic fever (DHF) is a kind of dengue fevers with more serious clinical symptoms like extremely high fever, severe hemorrhage, shock, low platelet count, pachyhematous, and high death rate [2]. A DHF accompanying with shock is called dengue shock syndrome (DSS).

*Aedes aegypti* and *Aedes albopictus* are two major vectors for transmission of dengue viruses [3]. Dengue fever is endemic in tropical and subtropical zones and has become one of the most wide-spread, infectious, and dangerous arboviral diseases. In recent years, there are several outbreaks of dengue fever and dengue hemorrhagic fever in Southeast Asia, Pacific Islands, Central America, and South America, making it a critical world-wide issue for public health [4].

Currently, there is no vaccine or antiviral treatment for dengue fever [5]. The patients are only recommended to drink plenty of fluids or take medicines like acetaminophen to avoid dehydration from vomiting and high fever [6]. Thus, it is very important to develop new antiviral drugs against dengue viruses. However, there are many difficulties in the synthesis of new compounds including high cost, low yields, and so on [7], which urges drug companies and research entities to search for antiviral drugs from natural products. It is known that traditional Chinese medicines are among the most studied natural products in curing diseases [8]. Owing to thousands of years' clinical trials of these medicines in China, traditional Chinese doctors have plenty of experience in combining natural products to cure diseases and protect people from the invasion of many viruses. Thus, it is promising to isolate antiviral drugs from Chinese medicinal herbs.

As a type of popular traditional Chinese medicinal herb, *Isatis tinctoria* (woad, Brassicaceae or Ban Lan Gen) has been proven to be effective against various viruses including influenza, hepatitis A virus (HAV), and so on [9–11]. It has also been used in treating the dengue fever with positive effects [12]. In this study, we isolated and purified the bioactive compounds of hydrosoluble constituents from the soil-growing woad by integrating bioassay-guided fraction technique and phytochemical isolation and purification techniques. In addition, we inferred the chemical structures of these compounds by polarimetry and 1D or 2D nuclear magnetic resonance (NMR).

## 2. Results

A total of 27 compounds, namely GB-1, GB-2, …, GB-27 were isolated from water-soluble components of aerial parts of *Isatis tinctoria*, and their structures were inferred as follows.

### 2.1. Structural Identification of Compound GB-1

**Figure 1:**
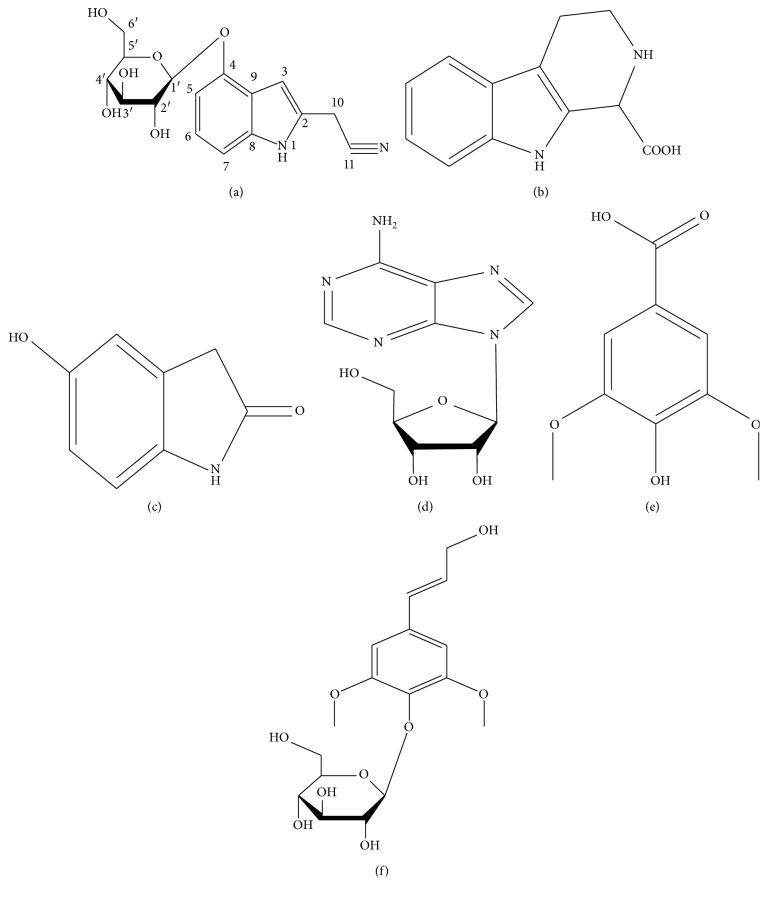
The structures of compounds (a) GB-1, (b) GB-2, (c) GB-3, (d) GB-4, (e) GB-5, and (f) GB-6.

Chemical compound GB-1 is a kind of white amorphous powder easily soluble in methanol, ethanol, and acetone. The identified chemical structure of GB-1 is plotted in Figure 1(a), and the detailed information about its ^1^H NMR (500 MHz, DMSO-*d*_6_) and ^13^C NMR (125 MHz, DMSO-*d*_6_) is listed in Table 1.

According to ^1^H NMR data, there exist four double-bond H signals including *δ*_H_ 7.19 (1H, s), *δ*_H_ 7.00 (1H, t, *J* = 8.6), *δ*_H_ 6.97(1H, d, *J* = 8.6), and *δ*_H_ 6.67 (1H, d, *J* = 8.6) in the low field. While in the relative high field, there exists H signal in AB system *δ*_H_ 4.17 (2H, d, *J* = 12.4), indicating the presence of methylene in the deshielded region. In addition, we also identified a set of glycon signals including: *δ*_H_ 4.88 (1H, d, *J* = 7.6), 3.25 (1H, dq, *J* = 9.0, 6.0), 3.72 (1H, dd, *J* = 12.0, 2.4), and 3.48 (1H, d, *J* = 12.0), which were inferred to be D-glucose.

As can be seen from ^13^C NMR data, there are eight C signals including *δ*_c_ 122.9, 120.3, 122.6, 103.3, 151.9, 106.1, 137.9, and 103.8 in the low field, indicating that the compound belongs to indole alkaloid. The methylene signal *δ*_c_ 14.9 is present in the high field, which also confirms the inference of the hydrogen spectrum. Combining with DEPT NMR, the compound contains 5 quaternary carbon signals, 9 methine signals, and 2 methylene signals. In addition, there is a group of glycon signals: *δ*_c_ 101.3, 77.1, 76.7, 73.5, 69.8, and 60.8. Thus, the monosaccharide can be inferred to be β-D-glucose. We also inferred other C and H signals by HSQC correlation analysis. According to the above NMR results, GB-1 is cappariloside A.

### 2.2. Structural Identification of Compound GB-2

Chemical compound GB-2 is a kind of bisque needle-like crystalloid, easily soluble in methanol and acetone. The identified chemical structure is plotted in Figure 1(b), and the detailed information about ^1^H NMR (500 MHz, DMSO-*d*_6_) and ^13^C NMR (125 MHz, DMSO-*d*_6_) is listed in Table S1. According to ^1^H NMR data, there exist four double-bond H signals including *δ*_H_ 7.46 (1H, d, *J* = 8.0), *δ*_H_ 7.33 (1H, d, *J* = 8.0), *δ*_H_ 7.08 (1H, t, *J* = 8.0), and *δ*_H_ 6.98 (1H, t, *J* = 8.0) in the low field, indicating the presence of *ortho*-disubstituted benzene ring structure. While in the relative high field region, there exist methylene H signal of *δ*_H_ 3.27 (1H, dd, *J* = 12.7, 4.0) and 3.01 (1H, dd, *J* = 12.7, 8.1).

As can be seen from ^13^C NMR data, there exist eight C signals, including *δ*_c_ 136.3, 126.3, 125.7, 121.7, 119.0, 117.8, 111.3, and 104.5 in the low field, suggesting that the compound belongs to indole alkaloid. The methylene signal of *δ*_c_ 14.9 and 40.1 is present in the high filed, which also confirms the inference of the hydrogen spectrum. Combining with the DEPT NMR, the compound contains 5 quaternary carbon signals, 5 methine signals, and 2 methylene signals. Thus, GB-2 was inferred to be 1-csrboline acid.

### 2.3. Structural Identification of Compound GB-3

Chemical compound GB-3 is a kind of bisque needle-like crystalloid, easily soluble in methanol and acetone. The identified chemical structure is plotted in Figure 1(c), and the detailed information about ^1^H NMR (500 MHz, DMSO-*d*_6_) and ^13^C NMR (125 MHz, DMSO-*d*_6_) is listed in Table S2. We compared the NMR data of GB-3 with that of GB-2, which suggests that GB-3 contains the signal of indole alkaloids. In addition, there exist the ABX system signal and the quaternary carbon signals of *δ*_c_ 152.3 in the H spectrum, indicating that hydroxyl substitution occurs at position C-5. Thus, GB-3 was inferred to be 2H-indol-2-one, 1,3-dihydro-5-hydroxy.

### 2.4. Structural Identification of Compound GB-4

Chemical compound GB-4 is a kind of white crystals, easily soluble in methanol and acetone. The identified structure is plotted in Figure 1(d), and the detailed data about ^1^H NMR (500 MHz, DMSO-*d*_6_) and ^13^C NMR (125 MHz, DMSO-*d*_6_) are listed in Table S3. According to ^1^H NMR data, there exist four double-bond H signals including *δ*_H_ 8.34 (1H, s) and *δ*_H_ 8.13 (1H, s). We inferred that the signal of *δ*_H_ 7.37 (2H, s) belongs to amino-H, which may be connected to a benzene ring or double bonds, and the signal of *δ*_H_ 7.37 (1H, d, *J* = 6.5) belongs to ribose terminal group H. Thus, we conjectured that the compound is β configuration based on the coupling constant.

As can be seen from ^13^C NMR data, there are five C signals including *δ*_c_ 87.9, 85.9, 73.5, 70.7, and *δ*_c_ 61.7, indicating the presence of ribose in the chemical compound. In addition, the C signal that exists in the low field is adenine. Combining with the DEPT NMR, the compound contains 3 quaternary carbon signals, 6 methine signals, and 1 methylene signal. Thus, GB-4 is adenosine.

### 2.5. Structural Identification of Compound GB-5

Chemical compound GB-5 is a kind of bisque needle-like crystalloid, easily soluble in methanol and acetone. The identified chemical structure is plotted in Figure 1(e), and the detailed data about ^1^H NMR (500 MHz, CD_3_OD) and ^13^C NMR (125 MHz, CD_3_OD) are listed in Table S4. According to ^1^H NMR data, there exist four covalent bonds H signal of *δ*_H_ 7.30 (2H, s) and methoxy signal of *δ*_H_ 3.87 (6H, s), suggesting that the compound has benzene ring substituted type of symmetrical structure. As can be seen from ^13^C NMR data, there are signal of *δ*_c_ 170.0 and methoxy signal of *δ*_c_ 56.7, indicating the presence of carboxylic acid structure in the compound. Combining with the DEPT NMR, the compound contains 5 quaternary carbon signals, 2 methine, signals and 2 methylene signals. Thus, GB-5 is syringic acid.

### 2.6. Structural Identification of Compound GB-6

Chemical compound GB-6 is a kind of bisque needle-like crystalloid, easily soluble in methanol. The identified chemical structure is plotted in Figure 1(f), and the detailed data about ^1^H NMR (500 MHz, CD_3_OD) and ^13^C NMR (125 MHz, CD_3_OD) are listed in Table S5. We compared the compound with chemical GB-5, indicating that they are of the same structural skeleton. However, the compound has more signals compared to GB-5, such as a set of double-bond signals, a hydroxymethyl signal (*δ*_c_ 62.8), and a set of glucose signal (*δ*_c_ 104.9, 78.8, 78.5, 76.1, and 62.6). Based on DEPT NMR, the compound contains 4 quaternary carbon signals, 9 methine signals, 2 methylene signals, and 2 methyl signals. Thus, GB-6 is syringin.

### 2.7. Structural Identification of Compound GB-7

**Figure 2:**
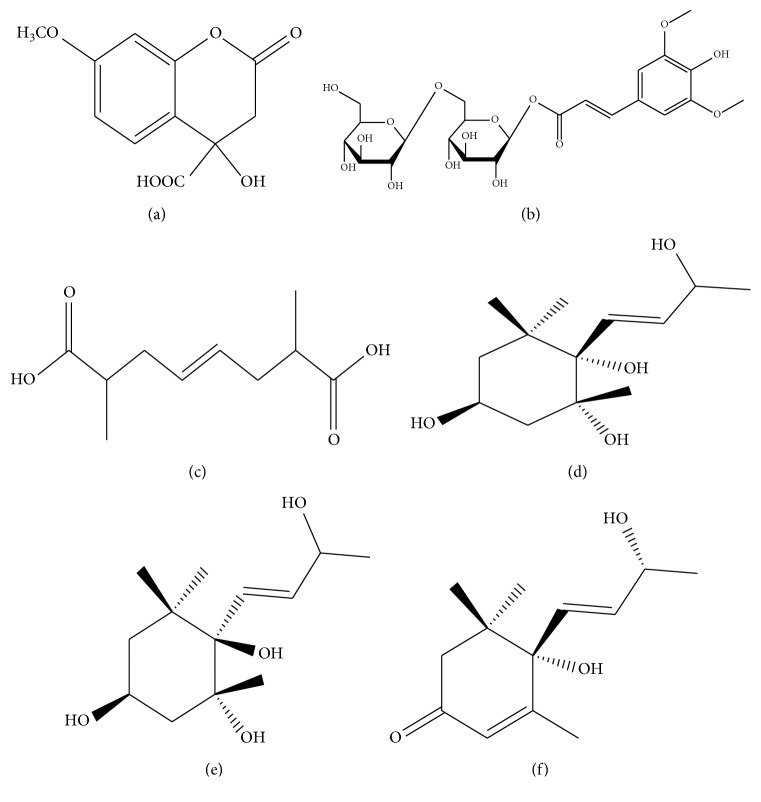
The structures of compounds (a) GB-7, (b) GB-8, (c) GB-9, (d) GB-10, (e) GB-11, and (f) GB-12.

Chemical compound GB-7 is a kind of white amorphous powder, easily soluble in methanol and acetone. The identified chemical structure is plotted in Figure 2(a), and the detailed data about ^1^H NMR (500 MHz, CD_3_OD) and ^13^C NMR (125 MHz, CD_3_OD) are listed in Table 2.

According to ^1^H NMR data, there are three H signals on benzene ring including *δ*_H_ 7.22 (1H, t, *J* = 7.8), *δ*_H_ 6.65 (1H, d, *J* = 7.8), and *δ*_H_ 6.60 (1H, d, *J* = 7.8), indicating that there exist benzene ring structure group *ortho*-substitution and also a methoxy H signal of *δ*_H_ 3.68 (3H, s).

As can be seen from ^13^C NMR data, there exist signal of *δ*_c_ 180.6 in the low field, indicating that the compound has carboxylic acid structure. In addition, there also exists a methoxy signal of *δ*_c_ 55.4. Combining with DEPT NMR, the compound contains 6 quaternary carbon signals, 3 methine signals, 1 methylene signal, and 1 methyl signal. However, we failed to find any compound that is consistent with these properties. After searching the SciFinder databases, we confirmed the compound GB-7 as a new monomer.

### 2.8. Structural Identification of Compound GB-8

Chemical compound GB-8 is a kind of bisque needle-like crystalloid, easily soluble in methanol and acetone. The identified chemical structure is plotted in Figure 2(b), and the detailed data about ^1^H NMR (500 MHz, CD_3_OD) and ^13^C NMR (125 MHz, CD_3_OD) are listed in Table S6. According to ^1^H NMR data, there exist an H signal on symmetrical spaces of benzene ring including *δ*_H_ 6.93 (2H, s) and double-bond signals *δ*_H_ 7.72 (1H, d, *J* = 15.8) and *δ*_H_ 6.44 (1H, d, *J* = 15.8), indicating that the double bonds possess trans configuration. In addition, there also exist two sets of end group signals of glycon including *δ*_H_ 4.33 (1H, d, *J* = 7.6) and 4.16 (1H, d, *J* = 7.2), which were inferred to be two D-glucose.

Besides the typical C signal, the ^13^C NMR data also contain two sets of signals of glycon including *δ*_c_ 95.8, 73.9, 77.9, 71.5, 77.9, and 69.5 and *δ*_c_ 104.5, 75.1, 77.7, 70.9, 77.8, and 62.6, indicating that the compound contains 2 glucose, which is connected by positions 1 and 6. Thus, GB-8 was inferred to be 1-(*O*-trans-3″,4″-dihydroxy-5″-methoxy)-*O*-trans-cinnamoyl gentiobiose.

### 2.9. Structural Identification of Compound GB-9

Chemical compound GB-9 is a kind of bisque oily matter, easily soluble in chloroform and ethyl acetate. The identified chemical structure is plotted in Figure 2(c), and the detailed data about ^1^H NMR (500 MHz, CD_3_OD) and ^13^C NMR (125 MHz, CD_3_OD) are listed in Table S7. According to ^1^H NMR data, there only exist five sets of H signals including *δ*_H_ 5.46 (1H, d, *J* = 15.6), *δ*_H_ 2.43 (1H, dd, *J* = 13.8, 6.8), 2.31 (1H, dt, *J* = 13.8, 4.6), and *δ*_H_ 2.12 (1H, dt, *J* = 13.8, 6.8). Since only one double-bond H signal is showed, the compound has a symmetrical structure with double bonds as the center.

As can be seen from ^13^C NMR data, there exists a signal of *δ*_c_ 180.1 in the low field, indicating that the compound has carboxylic acid structure. In addition, there also exist double bond signals, indicating the presence of a center double bond. Combining with the DEPT NMR, the compound contains 2 quaternary carbon signals, 4 methine signals, 2 methylene signals, and 2 methyl signals. As a result, GB-9 was inferred to be (E)-2, 7-dimethyloct-4-enedioic acid.

### 2.10. Structural Identification of Compound GB-10

Chemical compound GB-10 is a kind of white powder, easily soluble in methanol and chloroform. The identified chemical structure is plotted in Figure 2(d), and the detailed data about ^1^H NMR (500 MHz, CD_3_OD) and ^13^C NMR (125 MHz, CD_3_OD) are listed in Table S8. According to ^1^H NMR data, there exist 2 double bond H signals including *δ*_H_ 6.07 (1H, dd, *J* = 15.9, 3.3) and *δ*_H_ 5.77 (1H, dd, *J* = 15.9, 6.3) in the low field. While in the high field, there exist 4 methyl signals including *δ*_H_ 1.27 (3H, d, *J* = 6.0), *δ*_H_ 0.84 (3H, s), *δ*_H_ 1.14 (3H, s), and *δ*_H_ 1.20 (3H, s), suggesting the compound belongs to sesquiterpene type.

As can be seen from ^13^C NMR data, there exist signals of *δ*_C_ 136.2 and 131.3, indicating that the compound has a double bond structure. In addition, there also exist 4 methyl signals including *δ*_c_ 24.1, 26.2, 27.1, and 27.5. Combining with DEPT NMR, the compound contains 3 quaternary carbon signals, 4 methine signals, 2 methylene signals, and 4 methyl signals, indicating the compound to be lower 2 carbon sesquiterpenes. Thus, GB-10 is (3S, 5R, 6S)-6-((E)-3-hydroxybut-1-enyl)-1,1,5-trimethylcyclohexane-3,5,6-triol.

### 2.11. Structural Identification of Compound GB-11

Chemical compound GB-11 is a kind of white powder, easily soluble in methanol and chloroform. The identified chemical structure is plotted in Figure 2(e), and the detailed data about 1H NMR (500 MHz, CD_3_OD) and 13C NMR (125 MHz, CD_3_OD) are listed in Table S9. According to ^1^H NMR data, there exist two double bond H signals including *δ*_H_ 6.07 (1H, dd, *J* = 15.9, 3.3) and *δ*_H_ 5.77 (1H, dd, *J* = 15.9, 6.3) in the low field. While in the high field, there exist 4 methyl signals including *δ*_H_ 1.27 (3H, d, *J* = 6.0), *δ*_H_ 0.84 (3H, s), *δ*_H_ 1.14 (3H, s), and *δ*_H_ 1.20 (3H, s), suggesting that the compound belongs to sesquiterpene type.

As can be seen from ^13^C NMR data, there exist signals of *δ*_c_ 136.2 and 131.3 in the low field, indicating that the compound a has double bond structure. In addition, there also exist 4 methyl signals including *δ*_c_ 24.2, 26.2, 27.1, and 27.5. Combining with DEPT NMR, the compound contains 3 quaternary carbon signals, 4 methine signals, 2 methylene signals, and 4 methyl signals. In addition, there exist 13 C signals, and thus, the compound belongs to lower 2 carbon sesquiterpenes. Compared with the previous compound, only a difference was found in the spatial structure of position C-6. Thus, GB-11 was inferred to be (3S, 5R, 6R)-6-((E)-3-hydroxybut-1-enyl)-1,1,5-trimethylcyclohexane-3, 5,6-triol.

### 2.12. Structural Identification of Compound GB-12

Chemical compound GB-12 is a kind of white powder, easily soluble in methanol and chloroform. The identified chemical structure is plotted in Figure 2(f), and the detailed data about ^1^H NMR (500 MHz, CD_3_OD) and ^13^C NMR (125 MHz, CD_3_OD) are listed in Table S10. The NMR data of the GB-12 and GB-10 are quite similar. However, in the former, there exists an additional carbonyl signal of *δ*_c_ 201.1 in the low field along with a set of additional double-bond signals of *δ*_c_ 167.4 and 126.9, and 2 less methines, and 1 less methylene signal. Combining with DEPT NMR, the compound contains 4 quaternary carbon signals, 4 methine signals, 1 methylene signal, and 4 methyl signals. It is easily seen that there exist ^13^C signals, and thus the compound belongs to lower 2 carbon sesquiterpenes. As a result, GB-12 is (6S)-6-hydroxy-6-((E)-3-hydroxybut-1-enyl)-1,1,5-trimethylcyclohex-3-enone.

### 2.13. Structural Identification of Compound GB-13

**Figure 3:**
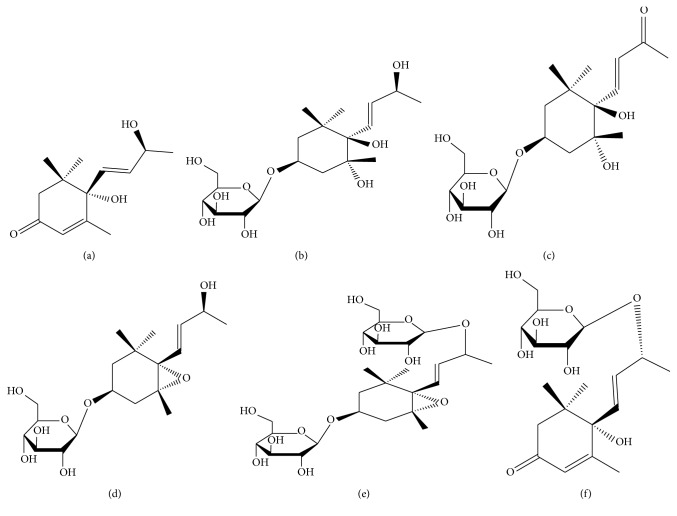
The structures of compounds (a) GB-13, (b) GB-14, (c) GB-15, (d) GB-16, (e) GB-17, and (f) GB-18.

Chemical compound GB-13 is a kind of white powder, easily soluble in methanol and chloroform. The identified chemical structure is plotted in Figure 3(a), and the detailed data about ^1^H NMR (500 MHz, CD_3_OD) and ^13^C NMR (125 MHz, CD_3_OD) are listed in Table S11. In general, the NMR data of GB-13 and GB-12 are very similar except for a difference in the spatial structure of C-9 position. Combining with DEPT NMR, the compound contains 4 quaternary carbon signals, 4 methine signals, 1 methylene signal, and 4 methyl signals. Thus, GB-13 is (6S)-6-hydroxy-6-((E)-3-hydroxybut-1-enyl)-1,1,5-trimethylcyclohex-3-enone.

### 2.14. Structural Identification of Compound GB-14

Chemical compound GB-14 is a kind of white powder, easily soluble in methanol and chloroform. The identified chemical structure is plotted in Figure 3(b), and the detailed data about 1H NMR (500 MHz, CD_3_OD) and 13C NMR (125 MHz, CD_3_OD) are listed in Table S12. The NMR data of the GB-14 and the GB-11 show that the compound skeleton was same and there are more glucose signals including C 102.0, 77.8, 77.7, 74.9, 71.4, and 62.5. Combining with DEPT NMR, the compound contains 3 quaternary carbon signals, 9 methine signals, 3 methylene signals, and 4 methyl signals, suggesting that there are 19 C signals, and the compound is a glycoside of lower 2 carbon sesquiterpenes. Thus, GB-14 was inferred to be (3S, 5R, 6R)-6-((E)-3-hydroxybut-1-enyl)-1,1,5-trimethylcyclohexane-5,6-diol 3-*O*-β-D-glucopyranosid.

### 2.15. Structural Identification of Compound GB-15

Chemical compound GB-15 is a kind of white powder, easily soluble in methanol and chloroform. The identified chemical structure is plotted in Figure 3(c), and the detailed data about 1H NMR (500 MHz, CD_3_OD) and 13C NMR (125 MHz, CD_3_OD) are listed in Table S13. The NMR data of the GB-15 and GB-14 show that the compound skeleton was the same, and there is only one more *δ*_C_ 200.3 carbonyl signal in the low field. Combining with DEPT NMR, the compound contains 4 quaternary carbon signals, 8 methine signals, 3 methylene signals, and 4 methyl signals, suggesting that there are 19 C signals, and the compound is a glycoside of lower 2 carbon sesquiterpenes. Thus, GB-15 is (3S, 5R, 6R)-6-((E)-3-one but-1-enyl)-1,1,5-trimethylcyclohexane-5,6-diol 3-*O*-β-D-glucopyranosid.

### 2.16. Structural Identification of Compound GB-16

Chemical compound GB-16 is a kind of white powder, easily soluble in methanol and chloroform. The identified chemical structure is plotted in Figure 3(d), and the detailed information is shown in Table S14. We compared compound GB-16 with compound GB-14 and found that they are of the same structural skeleton. In addition, GB-16 is only 18 molecular weight less than GB-14 and has no double bonds. It was speculated that the compound has an epoxy structure formed by two hydroxyl groups. Combining with DEPT NMR, the compound contains 3 quaternary carbon signals, 9 methine signals, 3 methylene signals, and 4 methyl signals, suggesting that there are 19 C signals, and the compound is a glycoside of lower 2 carbon sesquiterpenes. Thus, GB-16 was inferred to be (3S, 5R, 6S, 7E, 9S)-megastigman-7-ene-5,6-epoxy-3,9-diol 3-*O*-β-D-glucopyranoside.

### 2.17. Structural Identification of Compound GB-17

Chemical compound GB-17 is a kind of white amorphous powder, easily soluble in methanol. The identified chemical structure is plotted in Figure 3(e), and the detailed data about ^1^H NMR (500 MHz, CD_3_OD) and ^13^C NMR (125 MHz, CD_3_OD) are listed in Table S15. GB-17 has molecular weight of 162 more than GB-16, and there is one more set of glucose signals in NMR data, suggesting that GB-16 in turn connects to a glucose structure. Combining with DEPT NMR, the compound contains 3 quaternary carbon signals, 9 methine signals, 3 methylene signals, and 4 methyl signals, indicating that there are 19 C signals, and the compound is a glycoside of lower 2 carbon sesquiterpenes. Thus it was inferred to be (3S, 5R, 6S, 7E, 9S)-megastigman-7-ene-5,6-epoxy-3,9-diol 3,9-*O*-β-D-glucopyranoside.

### 2.18. Structural Identification of Compound GB-18

Chemical compound GB-18 is a kind of white amorphous powder, easily soluble in methanol. The identified chemical structure is plotted in Figure 3(f), and the detailed data are listed in Table S16. The NMR data of the GB-18 and the GB-12 show that they are of the same compound skeleton and GB-18 has only one more glucose signal including *δ*_c_ 102.9, 78.2, 77.9, 75.0, 71.7 and 62.8. Combining with DEPT NMR, the compound contains 3 quaternary carbon signals, 9 methine signals, 3 methylene signals, and 4 methyl signals, suggesting that there are 19 C signals and the compound is a glycoside of lower 2 carbon sesquiterpenes. Thus, GB-18 was inferred to be (6S)-6-hydroxy-6-((E)-3-α-hydroxybut-1-enyl)-1,1,5-trimethylcyclohex-3-enone 9-*O*-β-D-glucopyranosid.

### 2.19. Structural Identification of Compound GB-19

**Figure 4:**
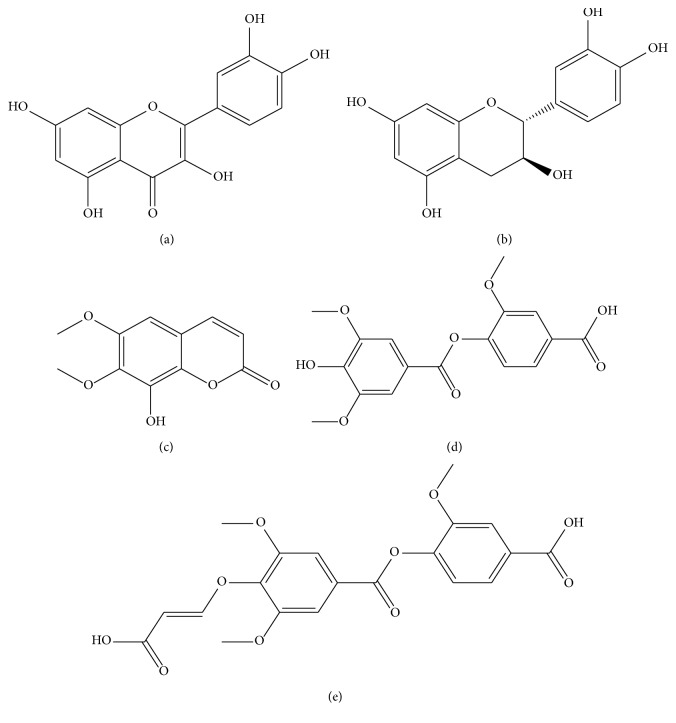
The structures of compounds (a) GB-19, (b) GB-20, (c) GB-21, (d) GB-22, and (e) GB-23.

Chemical compound GB-19 is a kind of bisque needle-like crystalloid, easily soluble in methanol. The identified chemical structure was plotted in Figure 4(a) and the detailed data are listed in Table S17. The ^1^H NMR of GB-19 has a H signal on the median of the benzene ring including *δ*_H_ 6.38 (1H, d, *J* = 1.6) and *δ*_H_ 6.17 (1H, d, *J* = 1.6), which indicating that all the compounds have A and C rings that are substituted by typical flavonoids C-2,3,5,7. There is a typical ABX system that exists in the B ring including *δ*_H_ 7.64 (1H, dd, *J* = 6.4, 1.6), *δ*_H_ 7.73 (1H, d, *J* = 1.6), and *δ*_H_ 6.87 (1H, d, *J* = 6.4), suggesting that the three substituted benzene ring structure is connected with C-2.

The ^13^C NMR data also confirm the inference of the H-spectrum. Combining with DEPT NMR, the compound GB-19 contains 3 quaternary carbon signals, and 9 methine signals. In the low field, there exist carbonyl C signal of *δ*_c_ 177.3, and 5 C signal connecting to the oxygen on the benzene ring including *δ*_C_ 165.7, *δ*_C_ 162.5, *δ*_C_ 158.2, *δ*_C_ 148.7, and *δ*_C_ 146.2. There also exist C signals of positions C-8 and C-6. As an indication, G-19 is quercetin.

### 2.20. Structural Identification of Compound GB-20

Chemical compound GB-20 is a kind of bisque needle-like crystalloid, easily soluble in methanol. The identified chemical structure is plotted in Figure 4(b), and the detailed information is listed in Table S18. By comparing with GB-19 and combining the results from the DEPT NMR, the compound contains 7 quaternary carbon signals, 7 methine signals, and 1 methylene signal, which was inferred to be catechin.

### 2.21. Structural Identification of Compound GB-21

Chemical compound GB-21 is a kind of bisque needle-like crystalloid, easily soluble in chloroform and acetone. The identified chemical structure is plotted in Figure 4(c), and the detailed data about ^1^H NMR (500 MHz, CDCl_3_) and ^13^C NMR (125 MHz, CDCl_3_) are listed in Table S19. According to ^1^H NMR data, there are 3 benzene ring H signals including *δ*_H_ 7.60 (1H, d, *J* = 9.6), *δ*_H_ 6.51 (1H, s), and *δ*_H_ 6.34 (1H, d, *J* = 9.6) in the low field. This is a typical H-spectrum signal of coumarin containing 2 methoxy signals, suggesting the compound to be three substituted coumarin types. Combining with the DEPT NMR, the compound contains 6 quaternary carbon signals, 3 methine signals, and 2 methyl signals. Thus, the above compound is fraxidin.

### 2.22. Structural Identification of Compound GB-22

Chemical compound GB-22 is a kind of bisque needle-like crystalloid, easily soluble in chloroform and acetone. The identified chemical structure is plotted in Figure 4(d), and the detailed data about ^1^H NMR (500 MHz, CDCl_3_) and ^13^C NMR (125 MHz, CDCl_3_) are listed in Table S20. There is an AMX system in the ^1^H NMR including *δ*_H_ 8.16 dd (8.0, 1.8, H-5′), *δ*_H_ 8.07 d (1.8, H-3′), and *δ*_H_ 7.3 d (8.3, H-6′). Based on the DEPT NMR data, 17 carbon signals are found in the carbon spectrum, and there are 9 quaternary carbon signals, 5 methine signals, and 3 methyl signals. Thus, GB-22 is eugenol oxalate.

### 2.23. Structural Identification of Compound GB-23

Chemical compound GB-23 is a kind of white powder, easily soluble in chloroform and acetone. The identified chemical structure is plotted in Figure 4(e), and the detailed data about ^1^H NMR (500 MHz, CDCl_3_) and ^13^C NMR (125 MHz, CDCl_3_) are listed in Table S21. The structure of compound GB-23 is similar to that of the compound GB-22, with the addition of a carboxylic acid signal and a set of double-bond signals. According to the DEPT NMR data, there are 10 quaternary carbon signals, 7 methine signals, and 3 methyl signals, which suggest that GB-23 is 4-*O*-acrylic-vanillic acid and 7-*O*-syringate.

### 2.24. Structural Identification of Compound GB-24

**Figure 5:**
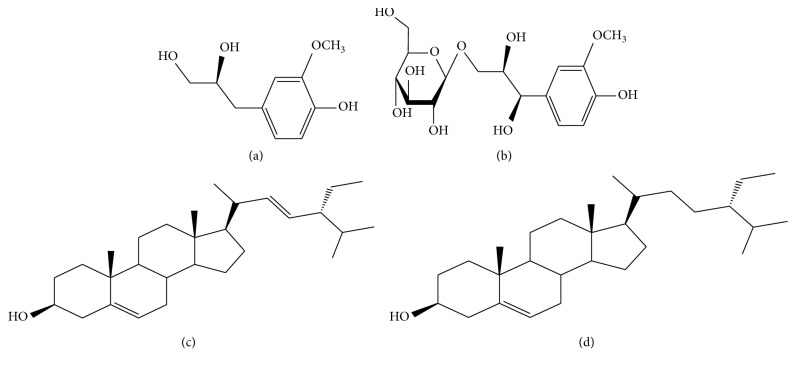
The structures of compounds (a) GB-24, (b) GB-25, (c) GB-26, and (d) GB-27.

Chemical compound GB-24 is a kind of white powder, easily soluble in chloroform and acetone. The identified chemical structure is plotted in Figure 5(a), and the detailed data about ^1^H NMR (500 MHz, CDCl_3_) and ^13^C NMR (125 MHz, CDCl_3_) are listed in Table S22.

According to the DEPT NMR data, there are 3 quaternary carbon signals, 4 methine signals, and 1 methyl signal. The methyl signal in this compound is *δ*_c_ 56.5, while the proton signal of the hydrogen spectrum is *δ*_H_ 3.85, indicating that the compound contains a methoxy group. As can be seen from the ^1^H NMR, there exists a set of signals of ABX system including *δ*_H_ 6.81 (1H, d, *J* = 2.0), 6.71 (1H, d, *J* = 8.0), and 6.65 (1H, dd, *J* = 8.0, 2.0), indicating that the compound contains a 1,3,4 three-substituted benzene ring. Thus, the compound is 3-(3,4-dihydroxyphenyl) propan-1,2-diol.

### 2.25. Structural Identification of Compound GB-25

Chemical compound GB-25 is a kind of white powder, easily soluble in methanol and chloroform. The identified chemical structure is plotted in Figure 5(b), and the detailed data about ^1^H NMR (500 MHz, CD_3_OD) and ^13^C NMR (125 MHz, CD_3_OD) are listed in Table S23. The compound GB-25 has additional one oxymethylene signal and a set of glucose signal compared to GB-24. Combining with the DEPT NMR, the compound contains 3 quaternary carbon signals, 10 methine signals, 1 methylene signal, and 1 methyl signal, which was inferred to be guaiacylglycerol 9-*O-*β-D-glucopyranoside.

### 2.26. Structural Identification of Compound GB-26 and GB-27

Chemical compound GB-26 and GB-27 are white powders, easily soluble in methanol and chloroform. The identified chemical structure is plotted in Figures 5(c) and 5(d), respectively. By comparing TLC on the H-spectrum data with those on the standard product, the compounds were inferred to be stigmasterol and β-sitosterol, respectively.

## 3. Discussion

Natural products are the treasures given by the nature, from which a great advantage in screening antiviral drug can be manifested. There is an inextricable link between the chemical studies of new structurally active natural products and the research on innovative drugs that are closely linked to human health. *Isatis tinctoria*, a traditional Chinese herbal medicine, has the effect of “clearing away heat and toxic material, cooling blood to eliminate plaque, treating fever, spotting, wind-heat embolism, and so on” [13]. The antiviral efficacy of *Isatis tinctoria* is mainly reflected in the treatment of acute infectious diseases. It has been reported that crude extraction of *Isatis tinctoria* has superior activity against influenza virus, hepatitis virus, haemorrhagic fever virus, human cytomegalovirus, coxsackievirus, herpes simplex virus, and endotoxin [14]. However, most of the current researches only focus on the research of crude extracts and mixtures, but less is on specific compounds. The antidengue ingredients of *Isatis tinctoria* have not been reported yet. Thus, we studied the chemical constituents of aerial parts of *Isatis tinctoria*, and a new compound GB-7 was obtained.

Existing studies have shown that the chemical composition of the *Isatis tinctoria* is a little bit complicated, and most studies focused on alkaloids, flavonoids, and lignans [15]. All these three compounds have excellent potential in biological activity, but the mechanisms of action of these compounds remain to be further discovered. In this work, based on tracking technology of antidengue virus activity, twenty-seven compounds, including one new compound (GB7), were isolated from the aerial parts of *Isatis tinctoria* purchased from Qiaocheng branch of the Bozhou herbs corporation in Anhui province, which is for the seek of laying the foundation for the later experiments. As be seen from the chemical structure, the compounds isolated from the aerial part of the *Isatis tinctorial* are mainly alkaloids, flavonoids, and phenolic compounds. Besides that, a series of 2-carbon-reduced sesquiterpenes, and their glycosides were obtained via separation technique, which are rarely reported in *Isatis tinctoria*. However, the biological activity of these compounds is still unclear. In the future, in vitro experiments including cytopathic effect (CPE) assay of C6/36 mosquito cells infected by the type 2 dengue virus (DV-2), MTT colorimetric assay, virus load test, and in vivo mouse infection experiments could be employed to validate the roles of GB-7 in treating dengue fever. In addition, the machine methods could be used to determine which compounds are the most important for the antiviral effects of *Isatis tinctoria*, as discussed in [16–18].

In order to obtain the active compound with antidengue virus and lower cytotoxicity quickly and effectively, combinatorial methods of bioactive trace technology and phytochemical extraction and separation are employed, which guide the isolation and purification of the effective chemical constituents on the water-soluble components of aerial parts of *Isatis tinctoria*, and identify its structure. In this way, a foundation for the development of plant-based antiviral drugs can be available.

## 4. Materials and Methods

### 4.1. Tested Plants

*Isatis tinctoria* was purchased from Qiaocheng branch of the Bozhou herbs corporation in Anhui province, China. After identification, drying, crushing, and over 40 mesh sieve, these tested plants were stored at room temperature for later use.

### 4.2. Main Instruments and Materials

The main instruments and materials used in this study were run following standard protocols including automatic tissue homogenizer, ultraviolet/visible spectrophotometer, ultra high speed refrigerated centrifuge, high-speed countercurrent chromatography, high-performance liquid chromatography, gas chromatography, nucleic acid extractor, fluorescent PCR instrument, ^1^H NMR (provided by Kunming Institute of Botany), ^13^C NMR (provided by Kunming Institute of Botany), Macroporous adsorption resin D101 (Dalian Elite Co., Ltd.), D201 (Dalian Elite Co., Ltd.), Sephadex LH-20 (Amersham Biotechnology, Switzerland), MCI (Mitsubishi Chemical, Japan), column chromatography silica gel, thin-layer chromatography silica gel, silica-gel plate (second branch of Qingdao Haiyang Chemical Co., Ltd.), RP18 (Dalian Elite Co., Ltd.), and EYELA rotary evaporator (Japan Chemical Co., Ltd).

### 4.3. Isolation of Components of Aerial Parts of Isatis tinctoria and Monomers

The processes of isolating various components and monomeric compounds from water-soluble components are as follows: (1) apply D101 for separation and purification (macroporous adsorption resin); (2) use MCI column (porous adsorption resin); (3) use D201 column (macroporous adsorption resin, D101 upgraded); (4) use D201 column; (5) use Sephadex LH20 column (hydroxypropyl cross-linked dextran); (6) use MCI column; and (7) use silica gel column.

### 4.4. Spectrum Analysis

Each purified sample was subjected to ^1^H NMR and ^13^C NMR spectra, and the pure compounds could be obtained after analyzing the spectrum.

## Figures and Tables

**Table 1:** ^1^H and ^13^C NMR data of compounds GB-1 in DMSO-*d*_6_ (*δ*, ppm; *J*, Hz).

Positions	^1^H NMR	^13^C NMR
2	7.19 (1H, s)	122.9 (CH)
3		120.3 (C)
4		122.6 (C)
5	6.67 (1H, d, *J* = 8.6)	103.3 (CH)
6	7.00 (1H, t, *J* = 8.6)	151.9 (CH)
7	6.97 (1H, d, *J* = 8.6)	106.1 (CH)
8		138.0 (C)
9		103.8 (C)
10	4.17 (2H, d, *J* = 12.4)	15.0 (CH_2_)
11		116.6 (C)
1′	4.88 (1H, d, *J* = 7.6)	101.3 (CH)
2′	3.60 (1H, dd, *J* = 9.0, 7.8)	73.6 (CH)
3′	3.91 (1H, dd, *J* = 9.0, 9.0)	76.7 (CH)
4′	3.44 (1H, t, *J* = 9.0)	69.8 (CH)
5′	3.25 (1H, dq *J* = 9.0, 6.0)	77.1 (CH)
6′	3.72 (1H, dd, *J* = 12.0, 2.4)	60.8 (CH_2_)
	3.48 (1H, d, *J* = 12.0)	

**Table 2:** ^1^H and ^13^C NMR data of compound GB-7 in DMSO-*d*_6_ (*δ*, ppm; *J*, Hz).

Positions	^1^H NMR	^13^C NMR
2		172.7 (C)
3	3.93 (2H, d, *J* = 14.7)	42.5 (CH_2_)
4		75.2 (C)
5	7.21 (1H, d, *J* = 7.6)	131.0 (CH)
6	6.60 (1H, br.d, *J* = 7.6)	105.8 (CH)
7		157.7 (C)
8	6.65 (1H, br.s)	103.8 (CH)
9		118.6 (C)
10		145.5 (C)
O-Me	3.68 (3H, s)	55.4 (CH_3_)
COOH		180.6 (C)

## Supplementary Materials

Supplementary Materials Table S1: ^1^H and ^13^C NMR data of compounds GB-2 in DMSO-*d*_6_ (*δ*, ppm; *J*, Hz). Table S2: ^1^H and ^13^C NMR data of compounds GB-3 in DMSO-*d*_6_ (*δ*, ppm; *J*, Hz). Table S3: ^1^H and ^13^C NMR data of compounds GB-4 in DMSO-*d*_6_ (*δ*, ppm; *J*, Hz). Table S4: ^1^H and ^13^C NMR data of compounds GB-5 in DMSO-*d*_6_ (*δ*, ppm; *J*, Hz). Table S5: ^1^H and ^13^C NMR data of compounds GB-6 in DMSO-*d*_6_ (*δ*, ppm; *J*, Hz). Table S6: ^1^H and ^13^C NMR data of compounds GB-8 in DMSO-*d*_6_ (*δ*, ppm; *J*, Hz). Table S7: ^1^H and ^13^C NMR data of compounds GB-9 in DMSO-*d*_6_ (*δ*, ppm; *J*, Hz). Table S8: ^1^H and ^13^C NMR data of compounds GB-10 in DMSO-*d*_6_ (*δ*, ppm; *J*, Hz). Table S9: ^1^H and ^13^C NMR data of compounds GB-11 in CD3OD (*δ*, ppm; *J*, Hz). Table S10: ^1^H and ^13^C NMR data of compounds GB-12 in CD3OD (*δ*, ppm; *J*, Hz). Table S11: ^1^H and ^13^C NMR data of compounds GB-13 in CD3OD (*δ*, ppm; *J*, Hz). Table S12: ^1^H and ^13^C NMR data of compounds GB-14 in CD3OD (*δ*, ppm; *J*, Hz). Table S13: ^1^H and ^13^C NMR data of compounds GB-15 in CD3OD (*δ*, ppm; *J*, Hz). Table S14: ^1^H and ^13^C NMR data of compounds GB-16 in CD3OD (*δ*, ppm; *J*, Hz). Table S15: ^1^H and ^13^C NMR data of compounds GB-17 in CD3OD (*δ*, ppm; *J*, Hz). Table S16: ^1^H and ^13^C NMR data of compounds GB-18 in CD3OD (*δ*, ppm; *J*, Hz). Table S17: ^1^H and ^13^C NMR data of compound GB-19 in CD3OD (*δ*, ppm; *J*, Hz). Table S18: ^1^H and ^13^C NMR data of compound GB-20 in CD3OD (*δ*, ppm; *J*, Hz). Table S19: ^1^H and ^13^C NMR data of compound GB-21 in CD3OD (*δ*, ppm; *J*, Hz). Table S20: ^1^H and ^13^C NMR data of compound GB-22 in CD3OD (*δ*, ppm; *J*, Hz). Table S21: ^1^H and ^13^C NMR data of compound GB-23 in CD3OD (*δ*, ppm; *J*, Hz). Table S22: ^1^H and ^13^C NMR data of compound GB-24 in CD3OD (*δ*, ppm; *J*, Hz). Table S23: ^1^H and ^13^C NMR data of compound GB-25 in CD3OD (*δ*, ppm; *J*, Hz).
