# Supplementary material for: Structure Analysis of Effective Chemical Compounds against Dengue Viruses Isolated from Isatis tinctoria
Source: Can J Infect Dis Med Microbiol. 2018 Apr 1;2018:3217473. doi: 10.1155/2018/3217473 (PMC5902107; doi:10.1155/2018/3217473)
Supplement: Supplementary Materials — Table S1: 1H and 13C NMR data of compounds GB-2 in DMSO-d 6 (δ, ppm; J, Hz). Table S2: 1H and 13C NMR data of compounds GB-3 in DMSO-d 6 (δ, ppm; J, Hz). Table S3: 1H and 13C NMR data of compounds GB-4 in DMSO-d 6 (δ, ppm; J, Hz). Table S4: 1H and 13C NMR data of compounds GB-5 in DMSO-d 6 (δ, ppm; J, Hz). Table S5: 1H and 13C NMR data of compounds GB-6 in DMSO-d 6 (δ, ppm; J, Hz). Table S6: 1H and 13C NMR data of compounds GB-8 in DMSO-d 6 (δ, ppm; J, Hz). Table S7: 1H and 13C NMR data of compounds GB-9 in DMSO-d 6 (δ, ppm; J, Hz). Table S8: 1H and 13C NMR data of compounds GB-10 in DMSO-d 6 (δ, ppm; J, Hz). Table S9: 1H and 13C NMR data of compounds GB-11 in CD3OD (δ, ppm; J, Hz). Table S10: 1H and 13C NMR data of compounds GB-12 in CD3OD (δ, ppm; J, Hz). Table S11: 1H and 13C NMR data of compounds GB-13 in CD3OD (δ, ppm; J, Hz). Table S12: 1H and 13C NMR data of compounds GB-14 in CD3OD (δ, ppm; J, Hz). Table S13: 1H and 13C NMR data of compounds GB-15 in CD3OD (δ, ppm; J, Hz). Table S14: 1H and 13C NMR data of compounds GB-16 in CD3OD (δ, ppm; J, Hz). Table S15: 1H and 13C NMR data of compounds GB-17 in CD3OD (δ, ppm; J, Hz). Table S16: 1H and 13C NMR data of compounds GB-18 in CD3OD (δ, ppm; J, Hz). Table S17: 1H and 13C NMR data of compound GB-19 in CD3OD (δ, ppm; J, Hz). Table S18: 1H and 13C NMR data of compound GB-20 in CD3OD (δ, ppm; J, Hz). Table S19: 1H and 13C NMR data of compound GB-21 in CD3OD (δ, ppm; J, Hz). Table S20: 1H and 13C NMR data of compound GB-22 in CD3OD (δ, ppm; J, Hz). Table S21: 1H and 13C NMR data of compound GB-23 in CD3OD (δ, ppm; J, Hz). Table S22: 1H and 13C NMR data of compound GB-24 in CD3OD (δ, ppm; J, Hz). Table S23: 1H and 13C NMR data of compound GB-25 in CD3OD (δ, ppm; J, Hz). [file 3217473.f1.docx]

**Table S1.** ^1^H and ^13^C NMR data of compounds GB-2 in DMSO-*d*_6_ (δ, ppm; *J*, Hz).

| **Positions** | **^1^H NMR** | **^13^C NMR** |
| --- | --- | --- |
| 2 |  | 126.3 (C) |
| 3 |  | 104.6 (C) |
| 4 | 7.33 (1H, d, *J* = 8.0) | 117.8 (C) |
| 5 | 7.08 (1H, t, *J* = 8.0) | 119.0 (CH) |
| 6 | 7.00 (1H, t, *J* = 8.0) | 121.7 (CH) |
| 7 | 7.45 (1H, d, *J* = 8.0) | 111.3 (CH) |
| 8 |  | 125.7 (C) |
| 9 |  | 136.3 (C) |
| 10 | 4.17 (2H, d, *J* = 12.4) | 21.9 (CH_2_) |
| 11 | 4.35 (2H, br.t, *J* = 12.4) | 40.1 (CH_2_) |
| 13 | 4.43 (1H, s) | 53.8 (CH) |

**Table S2.** ^1^H and ^13^C NMR data of compounds GB-3 in DMSO-*d*_6_ (δ, ppm; *J*, Hz).

| **Positions** | **^1^H NMR** | **^13^C NMR** |
| --- | --- | --- |
| 2 |  | 176.2 (CH) |
| 3 | 3.36 (2H, s) | 36.2 (CH_2_) |
| 4 | 6.58 (1H, d, *J* = 2.0) | 113.3 (CH) |
| 5 |  | 152.3 (C) |
| 6 | 6.53 (1H, br.d, *J* = 8.0) | 112.4 (CH) |
| 7 | 6.65 (1H, d, *J* = 8.0) | 109.5 (CH) |

**Table S3.** ^1^H and ^13^C NMR data of compounds GB-4 in DMSO-*d*_6_ (δ, ppm; *J*, Hz).

| **Positions** | **^1^H NMR** | **^13^C NMR** |
| --- | --- | --- |
| 2 | 8.34 (1H, s) | 140.0 (CH) |
| 4 |  | 156.2 (C) |
| 6 | 8.13 (1H, s) | 152.4 (CH) |
| 8 |  | 149.1 (C) |
| 9 |  | 119.4 (C) |
| 1′ | 5.86 (1H, d, *J* = 7.6) | 87.9 (CH) |
| 2′ | 4.59 (1H, t, *J* = 7.8) | 73.4 (CH) |
| 3′ | 4.13 (1H, d, *J* = 7.8) | 70.7 (CH) |
| 4′ | 3.95 (1H, d, *J* = 7.8) | 85.9 (CH) |
| 5′ | 3.66 (1H, dd, *J* = 12.0, 6.0)  3.53 (1H, dd, *J* = 12.0, 6.0) | 61.7 (CH_2_) |

**Table S4.** ^1^H and ^13^C NMR data of compounds GB-5 in DMSO-*d*_6_ (δ, ppm; *J*, Hz).

| **Positions** | **^1^H NMR** | **^13^C NMR** |
| --- | --- | --- |
| 1 |  | 121.8 (C) |
| 2,6 | 7.31 (2H, s) | 108.1 (CH) |
| 3,5 |  | 148.8 (C) |
| 4 |  | 141.6 (C) |
| O-Me | 3.87 (6H, s) | 56.7 (CH_3_) |
| COOH |  | 170.0 (C) |

**Table S5.** ^1^H and ^13^C NMR data of compounds GB-6 in DMSO-*d*_6_ (δ, ppm; *J*, Hz).

| **Positions** | **^1^H NMR** | **^13^C NMR** |
| --- | --- | --- |
| 1 |  | 134.0 (C) |
| 2,6 | 6.87 (2H, s) | 105.2 (CH) |
| 3,5 |  | 153.9 (C) |
| 4 |  | 132.6 (C) |
| 7 | 6.25 (1H, d, *J* = 15.8) | 131.2 (CH) |
| 8 | 5.81 (1H, dt, *J* = 15.8, 7.8) | 129.4 (CH) |
| 9 | 4.12 (2H, d, *J* = 7.8) | 62.8 (CH_2_) |
| O-Me | 3.73 (6H, s) | 56.6 (CH_3_) |
| 1′ | 4.88 (1H, d, *J* = 7.6) | 104.9 (CH) |
| 2′ | 3.60 (1H, dd, *J* = 9.0, 7.8) | 76.1 (CH) |
| 3′ | 3.91 (1H, dd, *J* = 9.0, 9.0) | 78.5 (CH) |
| 4′ | 3.44 (1H, t, *J* = 9.0) | 71.6 (CH) |
| 5′ | 3.25 (1H, dq *J* = 9.0, 6.0) | 78.8(CH) |
| 6′ | 3.72 (1H, dd, *J* = 12.0, 2.4)  3.48 (1H, d, *J* = 12.0) | 62.6 (CH_2_) |

**Table S6.** ^1^H and ^13^C NMR data of compounds GB-8 in DMSO-*d*_6_ (δ, ppm; *J*, Hz).

| **Positions** | **^1^H NMR** | **^13^C NMR** |
| --- | --- | --- |
| 1 |  | 126.4 (C) |
| 2,6 | 6.93 (2H, s) | 107.0 (CH) |
| 3,5 |  | 148.6 (C) |
| 4 |  | 139.9 (C) |
| 7 | 7.72 (1H, d, *J* = 15.8) | 149.5 (CH) |
| 8 | 6.43 (1H, d, *J* = 15.8) | 115.1 (CH) |
| 9 |  | 167.6 (C) |
| O-Me | 3.83 (6H, s) | 56.8 (CH_3_) |
| 1′ | 4.33 (1H, d, *J* = 7.6) | 95.8 (CH) |
| 2′ | 3.60 (1H, dd, *J* = 9.0, 7.8) | 76.1 (CH) |
| 3′ | 3.90 (1H, dd, *J* = 9.0, 9.0) | 78.5 (CH) |
| 4′ | 3.44 (1H, t, *J* = 9.0) | 71.6 (CH) |
| 5′ | 3.29 (1H, dq *J* = 9.0, 6.0) | 78.8(CH) |
| 6′ | 3.72 (1H, dd, *J* = 12.0, 2.4)  3.53 (1H, d, *J* = 12.0) | 69.5 (CH_2_) |
| 1′ | 4.11 (1H, d, *J* = 7.6) | 104.5 (CH) |
| 2′ | 3.58 (1H, dd, *J* = 9.0, 7.8) | 76.1 (CH) |
| 3′ | 3.91 (1H, dd, *J* = 9.0, 9.0) | 78.5 (CH) |
| 4′ | 3.44 (1H, t, *J* = 9.0) | 71.6 (CH) |
| 5′ | 3.25 (1H, dq *J* = 9.0, 6.0) | 78.8(CH) |
| 6′ | 3.72 (1H, dd, *J* = 12.0, 2.4)  3.48 (1H, d, *J* = 12.0) | 62.6 (CH_2_) |

**Table S7.** ^1^H and ^13^C NMR data of compounds GB-9 in DMSO-*d*_6_ (δ, ppm; *J*, Hz).

| **Positions** | **^1^H NMR** | **^13^C NMR** |
| --- | --- | --- |
| 1 |  | 180.1 (C) |
| 2 | 2.42 (1H, m) | 40.7 (CH) |
| 3 | 2.31 (1H, m)  2.11 (1H, m) | 37.7 (CH_2_) |
| 4 | 5.45 (1H, d, *J* = 17.6) | 130.7 (CH) |
| 5 | 1.11 (3H, d, *J* = 7.6) | 16.9 (CH_3_) |

**Table S8.** ^1^H and ^13^C NMR data of compounds GB-10 in DMSO-*d*_6_ (δ, ppm; *J*, Hz).

| **Positions** | **^1^H NMR** | **^13^C NMR** |
| --- | --- | --- |
| 1 |  | 40.7 (C) |
| 2 | 1.73 (1H, ddd, *J* = 13.1, 3.3, 1.4)  1.40 (1H, dd, *J* = 13.1,10.2) | 46.5 (CH_2_) |
| 3 | 3.90 (1H, m) | 65.3 (CH) |
| 4 | 2.41 (1H, ddd, *J* = 14.6, 5.0, 1.3)  1.80 (1H, dd, *J* = 14.6, 8.3) | 45.8 (CH_2_) |
| 5 | 7.21 (1H, d, *J* = 7.6) | 78.0 (C) |
| 6 |  | 79.0 (C) |
| 7 | 6.07 (1H, dd, *J* = 15.6, 3.3) | 131.2 (CH) |
| 8 | 5.77 (1H, dd, *J* = 15.6, 6.3) | 136.2 (CH) |
| 9 | 4.33 (1H, br.t, *J* = 6.0) | 69.0 (CH) |
| 10 | 1.27 (3H, d, *J* = 6.3) | 24.1 (CH_3_) |
| 11 | 0.84 (3H, s) | 27.5 (CH_3_) |
| 12 | 1.20 (3H, s) | 26.2 (CH_3_) |
| 13 | 1.14 (3H, s) | 27.1 (CH_3_) |

**Table S9.** ^1^H and ^13^C NMR data of compounds GB-11 in CD3OD (δ, ppm; J, Hz).

| **Positions** | **^1^H NMR** | **^13^C NMR** |
| --- | --- | --- |
| 1 |  | 40.7 (C) |
| 2 | 1.73 (1H, ddd, *J* = 13.1, 3.3, 1.4)  1.40 (1H, dd, *J* = 13.1,10.2) | 46.5 (CH_2_) |
| 3 | 3.90 (1H, m) | 65.3 (CH) |
| 4 | 2.41 (1H, ddd, *J* = 14.6, 5.0, 1.3)  1.80 (1H, dd, *J* = 14.6, 8.3) | 45.8 (CH_2_) |
| 5 | 7.21 (1H, d, *J* = 7.6) | 78.0 (C) |
| 6 |  | 78.5 (C) |
| 7 | 6.05 (1H, br.d, *J* = 15.6) | 131.2 (CH) |
| 8 | 5.77 (1H, dd, *J* = 15.6, 6.0) | 136.2 (CH) |
| 9 | 4.33 (1H, br.t, *J* = 6.0) | 69.0 (CH) |
| 10 | 1.33 (3H, d, *J* = 6.3) | 24.1 (CH_3_) |
| 11 | 0.95 (3H, s) | 27.5 (CH_3_) |
| 12 | 1.20 (3H, s) | 26.2 (CH_3_) |
| 13 | 1.18 (3H, s) | 27.1 (CH_3_) |

**Table S10.** ^1^H and ^13^C NMR data of compounds GB-12 in CD3OD (δ, ppm; J, Hz).

| **Positions** | **^1^H NMR** | **^13^C NMR** |
| --- | --- | --- |
| 1 |  | 40.3 (C) |
| 2 | 1.75 (1H, ddd, *J* = 13.1, 3.3, 1.4)  1.44 (1H, dd, *J* = 13.1,10.2) | 50.6 (CH_2_) |
| 3 |  | 201.1 (C) |
| 4 | 5.87 (1H, s) | 126.9 (CH) |
| 5 |  | 167.4 (C) |
| 6 |  | 79.8 (C) |
| 7 | 6.05 (1H, br.d, *J* = 15.6) | 129.8 (CH) |
| 8 | 5.71 (1H, dd, *J* = 15.6, 6.0) | 136.7 (CH) |
| 9 | 4.33 (1H, br.t, *J* = 6.0) | 68.6 (CH) |
| 10 | 1.33 (3H, d, *J* = 6.3) | 23.4 (CH_3_) |
| 11 | 0.95 (3H, s) | 24.4 (CH_3_) |
| 12 | 0.97 (3H, s) | 23.7 (CH_3_) |
| 13 | 0.88 (3H, s) | 19.5 (CH_3_) |

**Table S11.** ^1^H and ^13^C NMR data of compounds GB-13 in CD3OD (δ, ppm; J, Hz).

| **Positions** | **^1^H NMR** | **^13^C NMR** |
| --- | --- | --- |
| 1 |  | 40.3 (C) |
| 2 | 1.75 (1H, ddd, *J* = 13.1, 3.3, 1.4)  1.54 (1H, dd, *J* = 13.1,10.2) | 50.6 (CH_2_) |
| 3 |  | 201.1 (C) |
| 4 | 5.87 (1H, s) | 126.9 (CH) |
| 5 |  | 167.4 (C) |
| 6 |  | 79.8 (C) |
| 7 | 6.09 (1H, br.d, *J* = 15.6) | 130.0 (CH) |
| 8 | 5.71 (1H, dd, *J* = 15.6, 6.0) | 136.7 (CH) |
| 9 | 4.27 (1H, br.t, *J* = 6.0) | 68.4 (CH) |
| 10 | 1.33 (3H, d, *J* = 6.3) | 23.4 (CH_3_) |
| 11 | 0.95 (3H, s) | 24.4 (CH_3_) |
| 12 | 0.97 (3H, s) | 23.7 (CH_3_) |
| 13 | 0.88 (3H, s) | 19.5 (CH_3_) |

**Table S12.** ^1^H and ^13^C NMR data of compounds GB-14 in CD3OD (δ, ppm; J, Hz).

| **Positions** | **^1^H NMR** | **^13^C NMR** |
| --- | --- | --- |
| 1 |  | 40.6 (C) |
| 2 | 1.73 (1H, ddd, *J* = 13.1, 3.3, 1.4)  1.40 (1H, dd, *J* = 13.1,10.2) | 44.3 (CH_2_) |
| 3 | 3.90 (1H, m) | 73.4 (CH) |
| 4 | 2.41 (1H, ddd, *J* = 14.6, 5.0, 1.3)  1.80 (1H, dd, *J* = 14.6, 8.3) | 42.8 (CH_2_) |
| 5 | 7.21 (1H, d, *J* = 7.6) | 78.0 (C) |
| 6 |  | 78.5 (C) |
| 7 | 6.05 (1H, br.d, *J* = 15.6) | 130.8 (CH) |
| 8 | 5.77 (1H, dd, *J* = 15.6, 6.0) | 136.0 (CH) |
| 9 | 4.33 (1H, br.t, *J* = 6.0) | 69.0 (CH) |
| 10 | 1.33 (3H, d, *J* = 6.3) | 24.2 (CH_3_) |
| 11 | 0.95 (3H, s) | 27.5 (CH_3_) |
| 12 | 1.20 (3H, s) | 26.2 (CH_3_) |
| 13 | 1.18 (3H, s) | 27.1 (CH_3_) |
| 1′ | 4.88 (1H, d, *J* = 7.6) | 102.0 (CH) |
| 2′ | 3.60 (1H, dd, *J* = 9.0, 7.8) | 76.1 (CH) |
| 3′ | 3.91 (1H, dd, *J* = 9.0, 9.0) | 77.9 (CH) |
| 4′ | 3.44 (1H, t, *J* = 9.0) | 71.6 (CH) |
| 5′ | 3.25 (1H, dq *J* = 9.0, 6.0) | 79.1 (CH) |
| 6′ | 3.72 (1H, dd, *J* = 12.0, 2.4)  3.48 (1H, d, *J* = 12.0) | 62.6 (CH_2_) |

**Table S13.** ^1^H and ^13^C NMR data of compounds GB-15 in CD3OD (δ, ppm; J, Hz).

| **Positions** | **^1^H NMR** | **^13^C NMR** |
| --- | --- | --- |
| 1 |  | 36.0 (C) |
| 2 | 1.93 (1H, ddd, *J* = 13.1, 3.3, 1.4)  1.48 (1H, dd, *J* = 13.1,10.2) | 45.3 (CH_2_) |
| 3 | 3.96 (1H, m) | 73.4 (CH) |
| 4 | 2.43 (1H, ddd, *J* = 14.6, 5.0, 1.3)  1.80 (1H, dd, *J* = 14.6, 8.3) | 38.2 (CH_2_) |
| 5 |  | 68.0 (CH_2_) |
| 6 |  | 71.5 (C) |
| 7 | 6.05 (1H, br.d, *J* = 15.6) | 145.4 (CH) |
| 8 | 5.77 (1H, dd, *J* = 15.6, 6.0) | 133.8 (CH) |
| 9 |  | 200.3 (C) |
| 10 | 1.33 (3H, d, *J* = 6.3) | 27.2 (CH_3_) |
| 11 | 0.95 (3H, s) | 25.5 (CH_3_) |
| 12 | 1.20 (3H, s) | 29.2 (CH_3_) |
| 13 | 1.18 (3H, s) | 20.1 (CH_3_) |
| 1′ | 4.88 (1H, d, *J* = 7.6) | 102.9 (CH) |
| 2′ | 3.60 (1H, dd, *J* = 9.0, 7.8) | 76.1 (CH) |
| 3′ | 3.91 (1H, dd, *J* = 9.0, 9.0) | 77.9 (CH) |
| 4′ | 3.44 (1H, t, *J* = 9.0) | 71.6 (CH) |
| 5′ | 3.25 (1H, dq *J* = 9.0, 6.0) | 79.1 (CH) |
| 6′ | 3.72 (1H, dd, *J* = 12.0, 2.4)  3.48 (1H, d, *J* = 12.0) | 62.6 (CH_2_) |

**Table S14.** ^1^H and ^13^C NMR data of compounds GB-16 in CD3OD (δ, ppm; J, Hz).

| **Positions** | **^1^H NMR** | **^13^C NMR** |
| --- | --- | --- |
| 1 |  | 35.9 (C) |
| 2 | 1.73 (1H, ddd, *J* = 13.1, 3.3, 1.4)  1.40 (1H, dd, *J* = 13.1,10.2) | 45.3 (CH_2_) |
| 3 | 3.90 (1H, m) | 73.4 (CH) |
| 4 | 2.41 (1H, ddd, *J* = 14.6, 5.0, 1.3)  1.80 (1H, dd, *J* = 14.6, 8.3) | 38.5 (CH_2_) |
| 5 | 7.21 (1H, d, *J* = 7.6) | 67.0 (C) |
| 6 |  | 71.5 (C) |
| 7 | 6.05 (1H, br.d, *J* = 15.6) | 125.8 (CH) |
| 8 | 5.77 (1H, dd, *J* = 15.6, 6.0) | 139.0 (CH) |
| 9 | 4.33 (1H, br.t, *J* = 6.0) | 69.0 (CH) |
| 10 | 1.33 (3H, d, *J* = 6.3) | 24.0 (CH_3_) |
| 11 | 0.95 (3H, s) | 25.5 (CH_3_) |
| 12 | 1.20 (3H, s) | 29.2 (CH_3_) |
| 13 | 1.18 (3H, s) | 20.1 (CH_3_) |
| 1′ | 4.42 (1H, d, *J* = 7.6) | 103.0 (CH) |
| 2′ | 3.67 (1H, dd, *J* = 9.0, 7.8) | 75.1 (CH) |
| 3′ | 3.99 (1H, dd, *J* = 9.0, 9.0) | 77.9 (CH) |
| 4′ | 3.45 (1H, t, *J* = 9.0) | 71.4 (CH) |
| 5′ | 3.24 (1H, dq *J* = 9.0, 6.0) | 78.2 (CH) |
| 6′ | 3.72 (1H, dd, *J* = 12.0, 2.4)  3.48 (1H, d, *J* = 12.0) | 62.8 (CH_2_) |

**Table S15.** ^1^H and ^13^C NMR data of compounds GB-17 in CD3OD (δ, ppm; J, Hz) .

| **Positions** | **^1^H NMR** | **^13^C NMR** |
| --- | --- | --- |
| 1 |  | 35.9 (C) |
| 2 | 1.73 (1H, ddd, *J* = 13.1, 3.3, 1.4)  1.40 (1H, dd, *J* = 13.1,10.2) | 45.3 (CH_2_) |
| 3 | 3.90 (1H, m) | 73.4 (CH) |
| 4 | 2.41 (1H, ddd, *J* = 14.6, 5.0, 1.3)  1.80 (1H, dd, *J* = 14.6, 8.3) | 38.5 (CH_2_) |
| 5 | 7.21 (1H, d, *J* = 7.6) | 67.0 (C) |
| 6 |  | 71.5 (C) |
| 7 | 6.05 (1H, br.d, *J* = 15.6) | 125.8 (CH) |
| 8 | 5.77 (1H, dd, *J* = 15.6, 6.0) | 139.0 (CH) |
| 9 | 4.33 (1H, br.t, *J* = 6.0) | 77.4 (CH) |
| 10 | 1.33 (3H, d, *J* = 6.3) | 24.0 (CH_3_) |
| 11 | 0.95 (3H, s) | 25.5 (CH_3_) |
| 12 | 1.20 (3H, s) | 29.2 (CH_3_) |
| 13 | 1.18 (3H, s) | 20.1 (CH_3_) |
| 1′ | 4.42 (1H, d, *J* = 7.6) | 103.0 (CH) |
| 2′ | 3.68 (1H, dd, *J* = 9.0, 7.8) | 75.1 (CH) |
| 3′ | 3.99 (1H, dd, *J* = 9.0, 9.0) | 77.9 (CH) |
| 4′ | 3.45 (1H, t, *J* = 9.0) | 71.4 (CH) |
| 5′ | 3.24 (1H, dq *J* = 9.0, 6.0) | 78.2 (CH) |
| 6′ | 3.72 (1H, dd, *J* = 12.0, 2.4)  3.48 (1H, d, *J* = 12.0) | 62.8 (CH_2_) |
| 1′ | 4.42 (1H, d, *J* = 7.6) | 102.7.0 (CH) |
| 2′ | 3.67 (1H, dd, *J* = 9.0, 7.8) | 75.2 (CH) |
| 3′ | 3.99 (1H, dd, *J* = 9.0, 9.0) | 77.8 (CH) |
| 4′ | 3.45 (1H, t, *J* = 9.0) | 71.5 (CH) |
| 5′ | 3.24 (1H, dq *J* = 9.0, 6.0) | 78.4 (CH) |
| 6′ | 3.72 (1H, dd, *J* = 12.0, 2.4)  3.48 (1H, d, *J* = 12.0) | 62.8 (CH_2_) |

**Table S16.** ^1^H and ^13^C NMR data of compounds GB-18 in CD3OD (δ, ppm; J, Hz).

| **Positions** | **^1^H NMR** | **^13^C NMR** |
| --- | --- | --- |
| 1 |  | 40.3 (C) |
| 2 | 1.75 (1H, ddd, *J* = 13.1, 3.3, 1.4)  1.44 (1H, dd, *J* = 13.1,10.2) | 50.6 (CH_2_) |
| 3 |  | 201.1 (C) |
| 4 | 5.87 (1H, s) | 126.9 (CH) |
| 5 |  | 167.4 (C) |
| 6 |  | 79.8 (C) |
| 7 | 6.05 (1H, br.d, *J* = 15.6) | 129.8 (CH) |
| 8 | 5.71 (1H, dd, *J* = 15.6, 6.0) | 133.7 (CH) |
| 9 | 4.33 (1H, br.t, *J* = 6.0) | 77.6 (CH) |
| 10 | 1.33 (3H, d, *J* = 6.3) | 21.4 (CH_3_) |
| 11 | 0.95 (3H, s) | 24.4 (CH_3_) |
| 12 | 0.97 (3H, s) | 23.7 (CH_3_) |
| 13 | 0.88 (3H, s) | 19.5 (CH_3_) |
| 1′ | 4.42 (1H, d, *J* = 7.6) | 102.7.0 (CH) |
| 2′ | 3.67 (1H, dd, *J* = 9.0, 7.8) | 75.2 (CH) |
| 3′ | 3.99 (1H, dd, *J* = 9.0, 9.0) | 77.8 (CH) |
| 4′ | 3.45 (1H, t, *J* = 9.0) | 71.5 (CH) |
| 5′ | 3.24 (1H, dq *J* = 9.0, 6.0) | 78.4 (CH) |
| 6′ | 3.72 (1H, dd, *J* = 12.0, 2.4)  3.48 (1H, d, *J* = 12.0) | 62.8 (CH_2_) |

**Table S17.** ^1^H and ^13^C NMR data of compound GB-19 in CD3OD (δ, ppm; J, Hz).

| **Positions** | **^1^H NMR** | **^13^C NMR** |
| --- | --- | --- |
| 2 |  | 148.0 (C) |
| 3 |  | 137.2 (C) |
| 4 |  | 177.3 (C) |
| 5 |  | 162.5 (C) |
| 6 | 6.17 (1H, d, *J* = 1.6) | 99.2 (CH) |
| 7 |  | 165.7 (C) |
| 8 | 6.38 (1H, d, *J* = 1.6) | 94.4 (CH) |
| 9 |  | 158.2 (C) |
| 10 |  | 104.5 (C) |
| 1′ |  | 124.1 (C) |
| 2′ | 7.73 (1H, d, *J* = 1.6) | 116.0 (CH) |
| 3′ |  | 146.2 (C) |
| 4′ |  | 148.7 (C) |
| 5′ | 6.87 (1H, d, *J* = 6.4) | 116.2 (CH) |
| 6′ | 7.64 (1H, dd, *J* = 6.4, 1.6) | 121.0 (CH) |

**Table S18.** ^1^H and ^13^C NMR data of compound GB-20 in CD3OD (δ, ppm; J, Hz).

| **Positions** | **^1^H NMR** | **^13^C NMR** |
| --- | --- | --- |
| 2 | 4.63 (1H, d, *J* = 7.2) | 82.7 (CH) |
| 3 | 4.04 (1H, dd, *J* = 12.8, 7.2) | 68.8 (CH) |
| 4 | 2.86 (1H, dd, *J* = 16.0, 12.8)  2.55 (1H, dd, *J* = 16.0, 7.2) | 28.4 (CH_2_) |
| 5 |  | 157.5 (C) |
| 6 | 6.17 (1H, d, *J* = 1.6) | 96.7 (CH) |
| 7 |  | 156.8 (C) |
| 8 | 6.38 (1H, d, *J* = 1.6) | 95.9 (CH) |
| 9 |  | 157.4 (C) |
| 10 |  | 101.5 (C) |
| 1′ |  | 132.1 (C) |
| 2′ | 7.73 (1H, d, *J* = 1.6) | 115.5 (CH) |
| 3′ |  | 146.2 (C) |
| 4′ |  | 146.7 (C) |
| 5′ | 6.87 (1H, d, *J* = 6.4) | 116.2 (CH) |
| 6′ | 7.64 (1H, dd, *J* = 6.4, 1.6) | 121.0 (CH) |

**Table S19.** ^1^H and ^13^C NMR data of compound GB-21 in CD3OD (δ, ppm; J, Hz).

| **Positions** | **^1^H NMR** | **^13^C NMR** |
| --- | --- | --- |
| 2 | 4.63 (1H, d, *J* = 7.2) | 82.7 (CH) |
| 3 | 4.04 (1H, dd, *J* = 12.8, 7.2) | 68.8 (CH) |
| 4 | 2.86 (1H, dd, *J* = 16.0, 12.8)  2.55 (1H, dd, *J* = 16.0, 7.2) | 28.4 (CH_2_) |
| 5 |  | 157.5 (C) |
| 6 | 6.17 (1H, d, *J* = 1.6) | 96.7 (CH) |
| 7 |  | 156.8 (C) |
| 8 | 6.38 (1H, d, *J* = 1.6) | 95.9 (CH) |
| 9 |  | 157.4 (C) |
| 10 |  | 101.5 (C) |
| 1′ |  | 132.1 (C) |
| 2′ | 7.73 (1H, d, *J* = 1.6) | 115.5 (CH) |
| 3′ |  | 146.2 (C) |
| 4′ |  | 146.7 (C) |
| 5′ | 6.87 (1H, d, *J* = 6.4) | 116.2 (CH) |
| 6′ | 7.64 (1H, dd, *J* = 6.4, 1.6) | 121.0 (CH) |

**Table S20.** ^1^H and ^13^C NMR data of compound GB-22 in CD3OD (δ, ppm; J, Hz).

| **Positions** | **^1^H NMR** | **^13^C NMR** |
| --- | --- | --- |
| 2 | 4.63 (1H, d, *J* = 7.2) | 82.7 (CH) |
| 3 | 4.04 (1H, dd, *J* = 12.8, 7.2) | 68.8 (CH) |
| 4 | 2.86 (1H, dd, *J* = 16.0, 12.8)  2.55 (1H, dd, *J* = 16.0, 7.2) | 28.4 (CH_2_) |
| 5 |  | 157.5 (C) |
| 6 | 6.17 (1H, d, *J* = 1.6) | 96.7 (CH) |
| 7 |  | 156.8 (C) |
| 8 | 6.38 (1H, d, *J* = 1.6) | 95.9 (CH) |
| 9 |  | 157.4 (C) |
| 10 |  | 101.5 (C) |
| 1′ |  | 132.1 (C) |
| 2′ | 7.73 (1H, d, *J* = 1.6) | 115.5 (CH) |
| 3′ |  | 146.2 (C) |
| 4′ |  | 146.7 (C) |
| 5′ | 6.87 (1H, d, *J* = 6.4) | 116.2 (CH) |
| 6′ | 7.64 (1H, dd, *J* = 6.4, 1.6) | 121.0 (CH) |

**Table S21.** ^1^H and ^13^C NMR data of compound GB-23 in CD3OD (δ, ppm; J, Hz).

| **Positions** | **^1^H NMR** | **^13^C NMR** |
| --- | --- | --- |
| 2 | 4.63 (1H, d, *J* = 7.2) | 82.7 (CH) |
| 3 | 4.04 (1H, dd, *J* = 12.8, 7.2) | 68.8 (CH) |
| 4 | 2.86 (1H, dd, *J* = 16.0, 12.8)  2.55 (1H, dd, *J* = 16.0, 7.2) | 28.4 (CH_2_) |
| 5 |  | 157.5 (C) |
| 6 | 6.17 (1H, d, *J* = 1.6) | 96.7 (CH) |
| 7 |  | 156.8 (C) |
| 8 | 6.38 (1H, d, *J* = 1.6) | 95.9 (CH) |
| 9 |  | 157.4 (C) |
| 10 |  | 101.5 (C) |
| 1′ |  | 132.1 (C) |
| 2′ | 7.73 (1H, d, *J* = 1.6) | 115.5 (CH) |
| 3′ |  | 146.2 (C) |
| 4′ |  | 146.7 (C) |
| 5′ | 6.87 (1H, d, *J* = 6.4) | 116.2 (CH) |
| 6′ | 7.64 (1H, dd, *J* = 6.4, 1.6) | 121.0 (CH) |

**Table S22.** ^1^H and ^13^C NMR data of compound GB-24 in CD3OD (δ, ppm; J, Hz).

| **Positions** | **^1^H NMR** | **^13^C NMR** |
| --- | --- | --- |
| 2 | 4.63 (1H, d, *J* = 7.2) | 82.7 (CH) |
| 3 | 4.04 (1H, dd, *J* = 12.8, 7.2) | 68.8 (CH) |
| 4 | 2.86 (1H, dd, *J* = 16.0, 12.8)  2.55 (1H, dd, *J* = 16.0, 7.2) | 28.4 (CH_2_) |
| 5 |  | 157.5 (C) |
| 6 | 6.17 (1H, d, *J* = 1.6) | 96.7 (CH) |
| 7 |  | 156.8 (C) |
| 8 | 6.38 (1H, d, *J* = 1.6) | 95.9 (CH) |
| 9 |  | 157.4 (C) |
| 10 |  | 101.5 (C) |
| 1′ |  | 132.1 (C) |
| 2′ | 7.73 (1H, d, *J* = 1.6) | 115.5 (CH) |
| 3′ |  | 146.2 (C) |
| 4′ |  | 146.7 (C) |
| 5′ | 6.87 (1H, d, *J* = 6.4) | 116.2 (CH) |
| 6′ | 7.64 (1H, dd, *J* = 6.4, 1.6) | 121.0 (CH) |

**Table S23.** ^1^H and ^13^C NMR data of compound GB-25 in CD3OD (δ, ppm; J, Hz).

| **Positions** | **^1^H NMR** | **^13^C NMR** |
| --- | --- | --- |
| 2 | 4.63 (1H, d, *J* = 7.2) | 82.7 (CH) |
| 3 | 4.04 (1H, dd, *J* = 12.8, 7.2) | 68.8 (CH) |
| 4 | 2.86 (1H, dd, *J* = 16.0, 12.8)  2.55 (1H, dd, *J* = 16.0, 7.2) | 28.4 (CH_2_) |
| 5 |  | 157.5 (C) |
| 6 | 6.17 (1H, d, *J* = 1.6) | 96.7 (CH) |
| 7 |  | 156.8 (C) |
| 8 | 6.38 (1H, d, *J* = 1.6) | 95.9 (CH) |
| 9 |  | 157.4 (C) |
| 10 |  | 101.5 (C) |
| 1′ |  | 132.1 (C) |
| 2′ | 7.73 (1H, d, *J* = 1.6) | 115.5 (CH) |
| 3′ |  | 146.2 (C) |
| 4′ |  | 146.7 (C) |
| 5′ | 6.87 (1H, d, *J* = 6.4) | 116.2 (CH) |
| 6′ | 7.64 (1H, dd, *J* = 6.4, 1.6) | 121.0 (CH) |
